# Treatment of supraventricular arrhythmias in critical care patients with sepsis

**DOI:** 10.3389/fcvm.2026.1836476

**Published:** 2026-05-20

**Authors:** Tomas Tencer, Petr Waldauf, Martin Balik

**Affiliations:** 1 Department of Anaesthesiology, Resuscitation and Intensive Care Medicine, Motol and Homolka University Hospital and Second Faculty of Medicine, Charles University, Prague, Czechia; 2 Third Faculty of Medicine, Charles University, Prague, Czechia; 3 Department of Anaesthesiology and Resuscitation, Kralovske Vinohrady University Hospital, Third Faculty of Medicine, Charles University, Prague, Czechia; 4 Department of Anaesthesiology, Resuscitation and Intensive Care Medicine, General University Hospital and First Faculty of Medicine, Charles University, Prague, Czechia

**Keywords:** amiodarone, atrial fibrillation, beta-blockers, electrical cardioversion, landiolol, propafenone, septic shock, supraventricular arrhythmia

## Abstract

Supraventricular arrhythmias, especially new-onset atrial fibrillation, occur in approximately 8–46% of critically ill patients with sepsis, depending on the population and monitoring intensity. New-onset atrial fibrillation is independently associated with increased mortality, prolonged intensive care unit stay, and elevated thromboembolic risk. Sepsis-associated atrial fibrillation arises from the interplay of pre-existing patient risk factors, systemic inflammation, sepsis-induced atrial and ventricular remodeling, catecholamine exposure, mechanical ventilation, and electrolyte disturbances. Fluid loading further augments arrhythmia risk in the context of diastolic dysfunction and increased right-ventricular afterload in acute respiratory distress syndrome. Evidence regarding antiarrhythmic drug choice remains limited. The Propafenone vs. Amiodarone for Supraventricular Arrhythmias in Septic Shock trial, the only double-blind randomized comparison of these agents, showed similar 24-hour cardioversion rates but faster cardioversion and fewer recurrences with propafenone, with treatment effects strongly modified by left atrial volume index. Trials of ultra-short acting beta-blockers demonstrated reliable heart-rate control but heterogeneous and fragile mortality signals, with the suggested benefit largely driven by single-center esmolol data and not consistently reproduced in multicenter landiolol studies. Evidence for digoxin and magnesium supports primarily adjunctive roles rather than first-line rate- or rhythm-control strategies. No study has evaluated vernakalant specifically in sepsis, limiting the evidence for its use in hemodynamically unstable patients. Anticoagulation decisions in sepsis-associated new-onset atrial fibrillation are complicated by high bleeding risk, potential changes in stroke risk over time, and limited data on long-term thromboembolic outcomes, underscoring the need for individualized assessment. Overall, current evidence supports prioritizing correction of triggers and individualized selection of a rate- vs. rhythm-control strategy based on hemodynamic status, ventricular function, left atrial size, and arrhythmia characteristics. Future multicenter randomized trials are required to refine antiarrhythmic and anticoagulation strategies in this high-risk population.

## 1 Introduction

Sepsis and septic shock represent life-threatening organ dysfunction caused by a dysregulated host response to infection, affecting millions of patients worldwide and accounting for substantial ICU mortality (1). Among the numerous cardiovascular complications that complicate sepsis management, supraventricular arrhythmias (SVAs), particularly new-onset atrial fibrillation (NOAF), have emerged as both common and clinically consequential (2). The incidence of NOAF in critically ill patients varies widely across studies, ranging from 8% to 46% depending on the patient population, diagnostic criteria, severity of disease and monitoring methods employed (3–6).

Atrial fibrillation (AF) in critically ill patients is associated with a two- to fivefold increase in mortality compared to patients who maintain sinus rhythm (3, 7). The clinical importance of NOAF extends beyond the immediate hemodynamic consequences of the arrhythmia itself. NOAF in critically ill patients serves as both a marker of disease severity and an independent predictor of adverse outcomes, including increased mortality, prolonged ICU and hospital length of stay, higher healthcare costs, and increased risk of stroke (8, 9).

Despite the high incidence and prognostic impact of supraventricular arrhythmias in sepsis, optimal management remains uncertain, with major gaps in evidence regarding drug selection, timing of intervention, and anticoagulation strategies in critically ill patients. This review synthesizes current evidence on the epidemiology, pathophysiology, precipitating factors, and pharmacological management of SVAs in septic patients. We focus on trial data published through early 2026, with emphasis on how hemodynamic status and echocardiographic assessment should guide individualized treatment decisions at the bedside.

## 2 Pathophysiological mechanisms

The pathophysiology of NOAF in sepsis is multifactorial, involving a complex interplay of systemic inflammation, autonomic dysregulation, metabolic derangements, and structural cardiac changes (10).

Autonomic dysfunction in sepsis, characterized by increased sympathetic tone and reduced parasympathetic activity, contributes significantly to arrhythmogenesis. The combination of elevated catecholamine levels (both endogenous and exogenously administered) and altered autonomic balance creates conditions favorable for triggered activity and enhanced automaticity in atrial tissue (11, 12).

### 2.1 Causative factors for arrhythmias in septic patients

The development of supraventricular arrhythmias in septic patients results from a confluence of multiple predisposing factors that can be broadly categorized as patient-specific risk factors and precipitating condition and treatment-related factors (13).

The first category encompasses patient-specific predictors of NOAF. Advanced age represents one of the strongest independent patient-specific predictors in critically ill patients. The incidence of NOAF increases progressively in patients over 65 years (8, 9). Pre-existing cardiovascular disease, including coronary artery disease, heart failure, valvular disease, and hypertension, substantially increases the risk of AF development during critical illness (10, 14).

The second category comprises condition-related precipitating factors. Among these factors, the severity of sepsis and associated organ dysfunction correlates directly with NOAF incidence (4). Septic shock, characterized by profound hemodynamic instability, is associated with particularly high rates of NOAF (15). Hypoxemia and tissue hypoperfusion, common in severe sepsis, contribute to arrhythmia development through multiple mechanisms including myocardial ischemia and altered cellular metabolism (16, 17). Sepsis-induced myocardial dysfunction, manifested as both systolic and diastolic impairment, further increases susceptibility to atrial arrhythmias through elevated atrial pressures and wall stress (18).

The third category comprises treatment-related precipitating factors: hypovolemia, fluid resuscitation, vasopressor support, and mechanical ventilation may each contribute to NOAF through distinct proarrhythmic mechanisms (13). Understanding these iatrogenic precipitants is essential for balancing effective sepsis management with arrhythmia risk minimization.

## 3 Clinical phenotypes of supraventricular arrhythmias in sepsis

Supraventricular arrhythmias in sepsis may present in heterogeneous hemodynamic contexts that differ in both underlying mechanisms and optimal management. Recognizing pragmatic clinical phenotypes at the bedside can help translate pathophysiology into rapid and structured decisions.

### 3.1 Hemodynamically unstable phenotype

In this phenotype, atrial fibrillation or other supraventricular tachyarrhythmias directly precipitate or aggravate hypotension, shock, or signs of organ hypoperfusion. The loss of atrial contribution to ventricular filling, shortening of diastolic time, and irregular RR intervals can markedly reduce cardiac output. This is particularly pronounced in patients with diastolic dysfunction or right ventricular impairment. In such cases, immediate rhythm control is prioritized, and synchronized electrical cardioversion is generally indicated without delay, with pharmacologic therapy used as an adjunct to support sinus rhythm and prevent early recurrences (19).

### 3.2 Compensatory tachycardia phenotype

In many patients with septic shock, tachycardia reflects a compensatory response to reduced stroke volume or profound vasodilation rather than a primary driver of hemodynamic instability. Heart rate elevation helps preserve cardiac output despite low systemic vascular resistance, low effective circulating volume, or both. In this phenotype, overly aggressive heart rate control may worsen tissue perfusion and unmask or exacerbate shock. Management should therefore focus first on correcting the underlying hemodynamic disturbances, including optimization of preload and afterload guided by echocardiography and hemodynamic parameters, appropriate vasopressor support, and prompt source control.

### 3.3 Maladaptive adrenergic phenotype

In a subset of patients, persistent supraventricular tachyarrhythmias are driven predominantly by excessive sympathetic activation and high catecholamine exposure, despite adequate optimization of preload and afterload. This maladaptive adrenergic state contributes to myocardial oxygen supply-demand imbalance, arrhythmogenesis, and progressive ventricular dysfunction. In carefully selected patients with preserved or only moderately reduced systolic function, cautious titration of ultra-short acting beta-blockers may help blunt adrenergic toxicity. In this phenotype, any rate-slowing intervention must be closely coupled to bedside hemodynamic assessment, with frequent reassessment to avoid precipitating hypotension or hypoperfusion.

These phenotypes frequently overlap and evolve over time; however, recognizing the dominant mechanism at a given moment is essential to guide an appropriate therapeutic strategy. This framework helps align rhythm- and rate-control decisions with the underlying physiology and provides the conceptual basis for the pragmatic treatment algorithm presented later in this review. Our pragmatic phenotypes align with the ESICM shock guidelines 2025, which underscore the existence of distinct hemodynamic and echocardiographic profiles in septic shock and the need for individualized, multimodal assessment of tissue perfusion (20). They are also consistent with recent randomized trial designs such as ANDROMEDA-SHOCK-2, which explicitly advocate clinical hemodynamic phenotyping in early septic shock (using pulse pressure, diastolic arterial pressure, fluid responsiveness, and echocardiography) to guide individualized resuscitation strategies (21).

## 4 Iatrogenic and modifiable triggers of arrhythmias

### 4.1 Fluid therapy vs. diastolic dysfunction

Adequate fluid resuscitation, a cornerstone of early sepsis management according to Surviving Sepsis Campaign guidelines (22), presents a therapeutic paradox in patients with underlying diastolic dysfunction, right ventricular pathology, or propensity for atrial arrhythmias. Adequate fluid administration, when combined with early vasopressor therapy, may be considered antiarrhythmogenic, as it helps maintain tissue perfusion and hemodynamic stability. On the other hand, excessive fluid loading can precipitate or exacerbate atrial fibrillation through multiple mechanisms (23).

Volume overload increases left atrial pressure and wall stress, promoting acute atrial stretch and dilation. This mechanical stretch triggers immediate electrophysiological changes including shortening of the atrial effective refractory period, increased heterogeneity of atrial refractoriness, and enhanced susceptibility to AF initiation (24). In patients with pre-existing diastolic dysfunction, even moderate fluid volumes, when administered rapidly, can result in disproportionate increases in left atrial pressure, reflecting the steep left ventricular pressure-volume relationship characteristic of impaired relaxation (25).

Sepsis-induced diastolic dysfunction is increasingly recognized as a common and clinically significant manifestation of septic cardiomyopathy (26). Several echocardiographic studies using tissue Doppler imaging have demonstrated that diastolic dysfunction occurs in 40%–60% of septic patients (27). The pathophysiology involves direct inflammatory injury to the myocardium, mitochondrial dysfunction, and alterations in calcium handling that impair myocardial relaxation (26). Previous meta-analyses suggest that sepsis-associated left ventricular diastolic dysfunction (LVDD) is linked to increased mortality (27). A more recent multicenter study (PRODIASYS) using standardized 2016 ASE/EACVI criteria found LVDD to be highly prevalent but not independently associated with 28-day mortality in septic shock (28).

The 2025 ASE guidelines for diastolic function assessment provide updated criteria applicable to critically ill patients, although cautious interpretation must account for the effects of mechanical ventilation, vasoactive medications, and altered loading conditions. Key echocardiographic parameters include mitral inflow patterns (E/A ratio), tissue Doppler velocities (e′), and left atrial volume index (LAVi) (29). The presence of elevated E/e′ ratio in septic patients indicates elevated left atrial pressure and is likely to identify individuals at increased susceptibility to AF development (30).

#### 4.1.1 Clinical implications for fluid management

The recognition of diastolic dysfunction in septic patients has important implications for fluid management strategies (28). A more conservative, goal-directed approach to fluid resuscitation, guided by dynamic markers of fluid responsiveness rather than static pressure measurements, may help minimize the risk of AF precipitation while maintaining adequate tissue perfusion. Point-of-care echocardiography can help identify patients with diastolic dysfunction and elevated filling pressures who may be particularly vulnerable to fluid overload and AF, thereby supporting individualized adjustment of fluid and vasopressor therapy (30, 31).

### 4.2 Potential impact of mechanical ventilation

Beyond fluid management, mechanical ventilation itself exerts independent cardiovascular effects that influence supraventricular arrhythmia risk in patients with severe sepsis and septic shock. Positive-pressure ventilation reduces venous return and left ventricular preload, which may be beneficial in volume-overloaded patients but can compromise cardiac output in hypovolemic states, increase vasopressor requirements, and thereby promote arrhythmogenesis. Cyclical changes in intrathoracic pressure generate phasic variations in atrial transmural pressure and may modulate atrial refractoriness and conduction (32).

Right-ventricular afterload rises with positive-pressure ventilation, particularly in acute respiratory distress syndrome (ARDS) and at higher PEEP, leading to interventricular septal shift, impaired left ventricular filling, and increased susceptibility to atrial tachyarrhythmias. Acute cor pulmonale in severe ARDS is associated with right-atrial dilation and a higher burden of supraventricular arrhythmias (33, 34). In moderate-to-severe ARDS, prone positioning can unload the right ventricle by improving oxygenation, lowering plateau pressures, and attenuating hypoxic pulmonary vasoconstriction. These effects reduce right-ventricular enlargement and septal dyskinesis and may secondarily decrease SVA propensity in selected patients (35).

The relationship between specific ventilator settings and AF incidence remains incompletely defined, but lung-protective ventilation with lower tidal volumes and plateau pressures appears to confer hemodynamic benefits beyond pulmonary protection (36). High tidal volumes and elevated plateau pressures, which exaggerate intrathoracic pressure swings and right-ventricular load, may theoretically increase AF risk, although definitive data are lacking (5). In addition to ventilatory factors, numerous pharmacological agents routinely used in sepsis management carry independent proarrhythmic potential.

### 4.3 Medication

#### 4.3.1 Proarrhythmic effects of commonly used ICU medications

Numerous medications routinely administered in the intensive care unit possess proarrhythmic properties that can precipitate or perpetuate supraventricular arrhythmias in susceptible patients (5). Catecholamine vasopressors and inotropes, essential for hemodynamic support in septic shock, are among the most important iatrogenic contributors to AF in critically ill patients.

Norepinephrine, the first-line vasopressor for septic shock (1), increases atrial automaticity and triggered activity through beta-1 adrenergic stimulation, though its proarrhythmic effects are generally less pronounced than dopamine (37). Dopamine, particularly at doses exceeding 5 µg/kg/min where beta-adrenergic effects predominate, has been linked to increased AF incidence and is generally avoided in critically ill patients due to the poor predictability of its effects (38). Dobutamine, a synthetic catecholamine with predominantly beta-1 agonist activity, frequently precipitates or exacerbates tachyarrhythmias and should be used cautiously in patients with AF or at high risk for its development (39).

Vasopressin, a non-catecholamine vasoconstrictor acting primarily via V1 receptors, can be used as an adjunct to reduce catecholamine exposure in septic shock. By allowing down-titration of norepinephrine and other vasopressors, vasopressin may mitigate catecholamine-related proarrhythmic effects in susceptible patients (40). A large meta-analysis of distributive shock trials reported that treatment with vasopressin, alone or in combination with catecholamines, was associated with a significantly lower risk of atrial fibrillation compared with catecholamine vasopressors alone (relative risk approximately 0.77) (41). This finding is further supported by randomized data from the Surviving Sepsis Campaign 2026 evidence synthesis, in which adjunctive vasopressin was associated with probably less atrial fibrillation compared with norepinephrine monotherapy (RR 0.66; 95% CI, 0.42–1.05; moderate certainty) (22).

#### 4.3.2 Other medications with arrhythmogenic potential

Corticosteroids, commonly administered in critically ill patients, have been implicated in AF development through mechanisms including fluid retention, electrolyte disturbances, and potential direct effects on atrial electrophysiology. The relationship between corticosteroid use and AF appears dose-dependent, with higher cumulative doses associated with greater risk, although most of the available data derive from non-septic or mixed medical populations rather than septic-shock cohorts (42).

Numerous other ICU medications can contribute to arrhythmia risk through QT interval prolongation, electrolyte disturbances, or direct electrophysiological effects. These include certain antibiotics (particularly fluoroquinolones and macrolides), antifungal agents (especially azoles), anticholinergic neuroleptic agents, and antidepressant drugs (43).

### 4.4 Electrolyte disturbances in sepsis

Electrolyte abnormalities are highly prevalent in critically ill septic patients and play a significant role in arrhythmogenesis. The maintenance of normal electrolyte concentrations, particularly potassium and magnesium, represents a fundamental component of arrhythmia prevention and management in the ICU (44).

#### 4.4.1 Potassium and magnesium

Potassium homeostasis is frequently disrupted in sepsis through multiple mechanisms including renal dysfunction, gastrointestinal losses, medication effects (diuretics, insulin, beta-agonists), and transcellular shifts associated with acid-base disturbances. Hypokalemia increases atrial automaticity, prolongs action potential duration, and enhances susceptibility to triggered activity, thereby promoting both the development and maintenance of atrial fibrillation. Rather than avoiding overt hypokalemia, current expert recommendations emphasize maintaining serum potassium within the upper part of the normal range in patients with, or at elevated risk for, atrial arrhythmias (45–47).

Magnesium deficiency is highly prevalent in critically ill patients, with reported prevalence exceeding 50% in some ICU populations. Hypomagnesemia facilitates AF through multiple mechanisms including effects on potassium channel function, calcium handling, and membrane potential stability (48).

#### 4.4.2 Magnesium for AF treatment and prevention

Beyond correction of hypomagnesemia, intravenous magnesium has been investigated as both a treatment for established AF and as prophylaxis against NOAF in critically ill patients. Its potential antiarrhythmic mechanisms include raising the threshold for myocardial electrical excitation, slowing atrioventricular conduction, and increasing sinus node recovery time (49).

A 2023 systematic review and meta-analysis by Enayati et al. evaluated intravenous magnesium for non-postoperative AF with rapid ventricular response across nine randomized trials (1,048 patients), none of which specifically enrolled septic or general ICU populations (50). When added to standard care, magnesium was associated with increased odds of both rate control (OR 1.87, 95% CI 1.13–3.11) and rhythm control (OR 1.45, 95% CI 1.04–2.03). However, these benefits disappeared in sensitivity analyses restricted to studies with predefined lockout periods for rescue therapy (rate control: OR 0.97, 95% CI 0.59–1.59; rhythm control: OR 1.07, 95% CI 0.68–1.70), suggesting that earlier estimates may have been inflated by delayed assessment, spontaneous cardioversion, or concomitant treatments. Given the absence of sepsis-specific data, magnesium should not be considered a primary cardioversion agent in septic AF but may serve as adjunctive therapy, particularly when hypomagnesemia is suspected or documented.

Regarding prophylaxis, Curran et al. conducted a 2023 systematic review and meta-analysis of magnesium for prevention of NOAF outside the cardiac surgery setting (48).

Five randomized trials (4,713 patients) showed no significant reduction in NOAF with prophylactic magnesium compared with placebo (OR 0.72, 95% CI 0.48–1.09). The overall certainty of evidence was graded as very low owing to risk of bias, a limited number of trials, and indirectness; no study evaluated prophylactic magnesium in unselected ICU or septic populations.

Beyond randomized trials, a large quasi-experimental ICU study using a fuzzy regression discontinuity design across 93 ICUs (171,727 admissions; 478,901 twenty-four-hour treatment windows) evaluated magnesium supplementation in patients with serum levels close to institutional treatment cutoffs (0.67–0.82 mmol/L) (51). Magnesium administration within 8 h of testing was not associated with a significant reduction in ventricular or supraventricular tachyarrhythmias over the subsequent 24 h (risk difference 0.1%, 95% CI −4.2 to 6.9), nor did it meaningfully affect hypotension or mortality. Importantly, magnesium substitution was a routine part of the PRASE trial protocol (52), achieving median levels of 1.10–1.15 mmol/L in both study groups, considerably higher than those in the Goulden study, where many patients near the decision boundary would still have been considered hypomagnesemic by routine ICU standards.

## 5 A pragmatic treatment algorithm for supraventricular arrhythmias in sepsis

**Figure 1:**
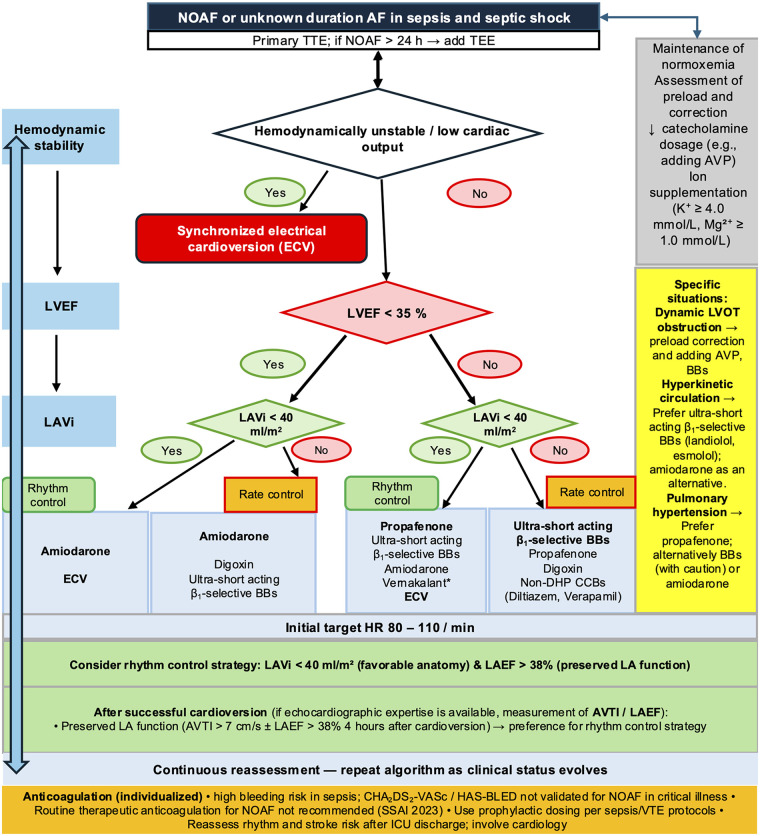
The Algorithm is adapted from the echocardiography-guided NOAF management approach proposed by Balik et al. for critically ill patients, tailored to sepsis and septic shock. Adapted from “Suggested algorithm for management of NOAF or AF of unknown duration, based on available intensive care medicine and cardiology data” by M. Balik, P. Vignon, M. S. Chew, G. Tavazzi, P. Mayo, G. Doufle, J. Aron, J. Hastings, B. Cholley, K. Jiroutkova, M. Slama, A. Herpain and A. McLean, licensed under CC BY-NC 4.0. The algorithm is structured around three sequential bedside assessments: hemodynamic stability, left ventricular ejection fraction and left atrial volume index. Synchronized electrical cardioversion is recommended as first-line treatment in hemodynamically unstable patients or those with low cardiac output (19). Correction of precipitating factors (preload optimization, reduction of catecholamine exposure, electrolyte supplementation, and maintenance of normoxemia) should be undertaken in parallel at all decision points. In hemodynamically stable patients, antiarrhythmic drug selection is guided by LVEF and LAVi: amiodarone is preferred in patients with LVEF < 35%, whereas propafenone is the first-line rhythm-control agent in patients with moderately reduced or preserved ventricular systolic function and non-dilated left atria (LAVi < 40 mL/m^2^). The sidebar summarizes drug preferences in specific clinical scenarios (hyperkinetic circulation, dynamic LVOT obstruction, and pulmonary hypertension) (104, 105). Anticoagulation should be individualized, recognizing the high bleeding risk in sepsis and the limited applicability of CHA_2_DS_2_-VASc and HAS-BLED scores in the ICU setting (97, 98); routine therapeutic anticoagulation for NOAF is not recommended per the 2023 SSAI guideline (53). After successful cardioversion, preserved left atrial function (AVTI > 7 cm ± LAEF > 38% at 4 h) supports a rhythm-control maintenance strategy (55). Vernakalant remains pharmacologically attractive for rhythm control given its atrial-selective mechanism and rapid onset; however, the absence of any sepsis-specific data and the drug's contraindication in patients with SBP < 100 mmHg restrict its applicability to a narrow subset of hemodynamically stable patients. AF, atrial fibrillation; AVTI, A-wave velocity-time integral on transmitral flow; AVP, arginine vasopressin; BBs, beta-blockers; CCBs, calcium channel blockers; CHA_2_DS_2_-VASc, congestive heart failure, hypertension, age ≥75, diabetes, stroke/TIA, vascular disease, age 65-74, sex category; DHP, dihydropyridine; ECV, electrical cardioversion; HAS-BLED, hypertension, abnormal renal/liver function, stroke, bleeding, labile INR, elderly, drugs/alcohol; HR, heart rate; LAEF, left atrial emptying fraction; LAVi, left atrial volume index; LVEF, left ventricular ejection fraction; LVOT, left ventricular outflow tract; NOAF, new-onset atrial fibrillation; SSAI, Scandinavian Society of Anaesthesiology and Intensive Care Medicine; TTE, transthoracic echocardiography; TEE, transesophageal echocardiography; VTE, venous thromboembolism.

The management of supraventricular arrhythmias in sepsis requires a structured, bedside-oriented approach that integrates hemodynamic status, arrhythmia characteristics, and echocardiographic findings (Figure 1). Current guidelines for AF management in the general population do not fully address the unique hemodynamic challenges of septic shock (19), while the 2023 SSAI guideline for NOAF in critically ill adults acknowledges very limited evidence to support any particular first-line pharmacological strategy (53). A recent state-of-the-art review has emphasized the need for individualized management structured around hemodynamic stability and cardiac function (7). To address this gap, a pragmatic, six-step treatment algorithm is proposed, grounded in the hemodynamic phenotypes described in Section 3, the reversible triggers reviewed in Section 4, and the echocardiographic framework developed from the PRASE trial and subsequent analyses (54–56).

### 5.1 Step 1: identify and treat hemodynamic instability

The first priority is to determine whether the supraventricular arrhythmia is the primary driver of hemodynamic instability. In patients with shock or severe hemodynamic instability directly attributable to the tachyarrhythmia, corresponding to the hemodynamically unstable phenotype (Section 3.1), immediate synchronized electrical cardioversion (ECV) is indicated (3, 19). In the 2024 ESC guidelines, emergency ECV carries a Class I recommendation and takes precedence over thrombus exclusion, which is addressed only after hemodynamic stabilization. However, the success of ECV in critically ill patients is limited, with fewer than 30% converting to sinus rhythm, and among those who initially convert, 40%–60% experience AF recurrence (7, 57). Accordingly, ECV should be combined with concomitant administration of an antiarrhythmic agent, which improves the success rate to above 80% and reduces the likelihood of early recurrence (57, 58). In the hemodynamically stable patient, a primary transthoracic echocardiographic (TTE) examination should be performed to guide further management (Steps 2–3).

### 5.2 Step 2: correct reversible triggers

Before or concurrent with initiating antiarrhythmic therapy, all modifiable triggers identified in Section 4 should be systematically addressed. This includes optimization of volume status guided by TTE echocardiographic assessment and dynamic markers of fluid responsiveness (Section 4.1), correction of hypoxemia and adjustment of ventilatory settings to minimize right ventricular afterload (Section 4.2), review and minimization of proarrhythmic medications, particularly high-dose catecholamines, with consideration of vasopressin as an adjunctive vasopressor to reduce catecholamine exposure (Section 4.3), and correction of electrolyte disturbances, with serum potassium targeted to ≥ 4.0 mmol/L and magnesium to ≥ 1.0 mmol/L (Section 4.4; Figure 1) (5, 7, 58). In many patients with NOAF, treatment of the underlying cause and correction of reversible factors results in spontaneous cardioversion without the need for additional pharmacological intervention (7).

### 5.3 Step 3: echocardiographic assessment

Point-of-care echocardiography plays a central role in guiding both treatment strategy and antiarrhythmic drug selection (58). The assessment follows a structured sequence corresponding to the decision points in Figure 1.

#### 5.3.1 Left ventricular systolic function

Left ventricular ejection fraction (LVEF) < 35% identifies patients in whom agents with significant negative inotropic properties, including propafenone, non-dihydropyridine calcium channel blockers, vernakalant, and beta-blockers, should be avoided (19, 58). In these patients, treatment is reserved for amiodarone and/or digoxin, which have minimal cardiodepressant effects and can be used safely in the setting of reduced systolic function (Figure 1). Intravenous amiodarone, digoxin, or ultra-short acting beta-blockers may also be considered in patients with AF who have hemodynamic instability or severely depressed LVEF to achieve acute control of heart rate (Class IIb, Level B) (19). Conversely, patients with preserved or only moderately reduced LVEF ≥ 35% are candidates for a wider range of pharmacological options, and the next echocardiographic decision point, left atrial size, guides further selection.

#### 5.3.2 Left atrial volume index

Left atrial volume index (LAVi) is the key parameter for distinguishing patients likely to benefit from rhythm control vs. those in whom rate control is more appropriate.

LAVi < 40 mL/m^2^ indicates favorable atrial anatomy and identifies patients who may benefit from a rhythm-control strategy, whereas LAVi exceeding 48 mL/m^2^ suggests significant atrial remodeling that reduces the likelihood of sustained sinus rhythm and favors a rate-control approach (58). In the intermediate range (40–48 mL/m^2^), the decision should be guided by additional functional parameters assessed after cardioversion (Step 6) and by the clinical context. Importantly, a moderately dilated LA should not preclude attempts at cardioversion in hemodynamically unstable patients with low cardiac output and NOAF (58).

### 5.4 Step 4: define rate control vs. rhythm control treatment strategy

Based on the echocardiographic assessment and clinical context, the treatment strategy is defined. The choice between rate and rhythm control should be individualized according to hemodynamic stability, duration of the arrhythmia, likelihood of successful cardioversion, and the echocardiographic phenotype identified at the bedside (3, 5, 7, 58). Major cardiology trials such as AFFIRM, RACE, AF-CHF have failed to demonstrate a benefit of rhythm control over rate control in the general AF population (59–61). However, these trials did not consider the degree of pre-existing ventricular systolic and diastolic dysfunction, which can be of paramount importance in the acutely decompensated patient, nor did they address the hemodynamic context of septic shock (58). In patients with septic shock, cardioversion to sinus rhythm was associated with an immediate improvement in stroke volume, a rise in systemic vascular resistance, and a consequent reduction in vasopressor requirements within 4 h (55, 58).

#### 5.4.1 Rhythm control

Rhythm control is favored when: (1) AF contributes to hemodynamic compromise despite adequate volume and vasopressor optimization; (2) the arrhythmia is of recent onset; (3) LAVi < 40 mL/m^2^, indicating favorable atrial anatomy; (4) restoration of atrial systole is considered important for cardiac output, particularly in patients with diastolic dysfunction characterized by impaired relaxation or a pseudonormalization pattern, where left ventricular filling is substantially dependent on active atrial contraction; or (5) right ventricular dysfunction or pulmonary hypertension is present (55, 58, 62).

#### 5.4.2 Rate control

Rate control is generally preferred when: (1) the arrhythmia is well tolerated hemodynamically; (2) it has been present for a prolonged or uncertain duration without possibility of TEE (transesophageal echocardiography); (3) LAVi exceeds 48 mL/m^2^, indicating significant atrial remodeling; (4) moderate-to-severe valvular disease is present; (5) high-dose catecholamine requirements persist; or (6) advanced diastolic dysfunction with a restrictive filling pattern is present, where rate control may be more appropriate than rhythm control (3, 58, 63). However, rate control does not restore the hemodynamic contribution of atrial systole, which may be particularly important in critically ill patients with compromised cardiac output.

When rate control is chosen, an initial target heart rate of 80–110 beats per minute is recommended as a pragmatic starting point (Figure 1), with subsequent adjustment guided by hemodynamic response and clinical tolerance. A lenient heart rate target of 110 beats per minute, as suggested for the non-critically ill population (Class IIa, Level B) (19), may not be appropriate for patients who rely on heart rate optimization to maintain adequate cardiac output (7). When rhythm control is chosen, current evidence supports its consideration for all patients with LAVi < 40 mL/m^2^ (favorable anatomy) and LAEF > 38% (preserved LA function) (Figure 1).

### 5.5 Step 5: pharmacological agent selection

Based on the hemodynamic and echocardiographic assessment, the specific pharmacological agent is selected. The drug lists in Figure 1 are ordered with the most preferred agents listed first for each clinical scenario.

#### 5.5.1 Amiodarone

Amiodarone, a class III antiarrhythmic, remains the most widely used antiarrhythmic agent in the ICU and is the preferred agent in patients with structural heart disease, reduced left ventricular function (LVEF < 35%), or significant atrial dilation (LAVi ≥ 40 mL/m^2^) (3, 7, 64). Its minimal cardiodepressant effect makes it uniquely suitable for patients with compromised ventricular function. Amiodarone provides both rate- and rhythm-control properties and is commonly combined with ECV, improving the cardioversion success rate to above 80% (2, 3, 65, 66). The PRASE trial demonstrated that amiodarone and propafenone achieved similar 24-hour cardioversion rates in septic shock patients with supraventricular arrhythmias, but amiodarone performed favorably and appears to remain the preferred agent in patients with dilated left atria (LAVi ≥ 40 mL/m^2^) (54, 56). In a separate retrospective ICU study, amiodarone achieved heart-rate control (<110 bpm) more rapidly than digoxin (median 2 vs. 4 h), at the expense of higher rates of bradycardia and increased vasopressor requirements, with approximately one quarter of patients having sepsis (67).

The adverse effect profile of amiodarone is extensive and includes hypotension, bradycardia, and QT interval prolongation (68, 69). Long-term adverse effects, including pulmonary toxicity, thyroid dysfunction, hepatotoxicity, and corneal deposits, are less frequent in the acute ICU setting, but become important considerations if prolonged therapy is anticipated (70–74).

#### 5.5.2 Propafenone

Propafenone is a class IC antiarrhythmic agent with potent sodium channel blockade as its primary mechanism of action, supplemented by mild beta-adrenergic blocking activity and minor calcium channel antagonism (5). Historically, the use of class IC agents in patients with structural heart disease has been viewed with caution owing to the results of the CAST trial, which demonstrated increased mortality with flecainide and encainide in post-myocardial infarction patients with ventricular arrhythmias (75). The CAST population comprised post-MI patients with ventricular ectopy and severely impaired LV function, a population that differs substantially from the carefully selected septic shock patients enrolled in the PRASE trial (54). Moreover, the CAST trial did not include any patients on propafenone therapy. Propafenone also differs pharmacologically from flecainide and encainide, with a shorter elimination half-life, weaker sodium-channel binding kinetics, and intrinsic beta-blocking activity, which may contribute to a different safety profile.

The PRASE trial (Propafenone vs. Amiodarone for Supraventricular Arrhythmias in Septic Shock) is the only prospective, double-blind, randomized trial to directly compare two pharmacological rhythm-control strategies in septic shock (54). Patients with LVEF < 35% were excluded as well as the patients supported by more than 1.0 µg/kg/min of noradrenaline. The primary endpoint, the proportion of patients achieving sinus rhythm at 24 h, was met by 72.8% in the propafenone group and 67.3% in the amiodarone group, a difference that did not reach statistical significance (*p* = 0.4). Despite the neutral primary outcome, several secondary analyses favored propafenone: time to successful cardioversion was significantly shorter (median 3.7 h vs. 7.3 h, *p* = 0.02), and propafenone was associated with markedly fewer arrhythmia recurrences (52% vs. 76%, *p* < 0.001).

A pre-specified subgroup analysis revealed that the treatment effect was substantially modified by left atrial size. In patients with a non-dilated left atrium (LAVi < 40 mL/m^2^, *n* = 133), propafenone was significantly superior to amiodarone for the primary endpoint (81.3% vs. 65.7%, *p* = 0.04), with faster cardioversion (median 3.6 vs. 8.5 h, *p* = 0.009) and improved survival at 1 month (*p* = 0.007) and 1 year (HR 0.60, *p* = 0.014). In contrast, patients with a dilated left atrium (LAVi ≥ 40 mL/m^2^) showed a trend toward worse outcomes with propafenone, including higher 1-month mortality (HR 3.6, *p* = 0.045) and slower cardioversion (median 18 h vs. 6.4 h with amiodarone, *p* = 0.05) (56). The PRASE trial establishes propafenone as a safe and effective rhythm-control agent in septic shock patients with preserved left atrial dimensions and preserved or moderately reduced LVEF. This trial highlights the critical importance of bedside echocardiographic assessment in guiding antiarrhythmic drug selection (58).

#### 5.5.3 Beta-blockers

Tachycardia is nearly universal in septic shock and may reflect either a compensatory response to low stroke volume or excessive adrenergic activation that contributes to myocardial injury, arrhythmogenesis, and increased oxygen consumption (5).

Ultra-short acting beta-blockers (esmolol, landiolol) may be considered for rate control in carefully selected patients with a hyperadrenergic, maladaptive phenotype (Section 3.3). Landiolol has demonstrated safe and effective heart rate control even in patients with acute decompensated heart failure (ADHF) due to atrial fibrillation (LVEF < 40%, median 22%, IQR 18%–32%) (76). Right heart catheterization confirmed hemodynamic improvement including reduced PCWP and maintained cardiac output, without serious adverse events. However, in a subgroup analysis, patients with more dilated left ventricles (LVEDVI ≥ 84 mL/m^2^) and lower mean blood pressure (≤ 97 mmHg) were at higher risk of adverse events, suggesting that the degree of ventricular remodeling and baseline hemodynamic reserve should be considered before initiating landiolol (76).

Although these data derive from a non-septic ADHF population, several randomized trials have evaluated these agents specifically in sepsis. The J-Land 3S trial demonstrated effective heart rate control with landiolol without significant impact on blood pressure in patients with sepsis-related tachyarrhythmia (77). The STRESS-L trial raised safety concerns, reporting higher organ dysfunction scores with landiolol, although the study enrolled patients with septic shock irrespective of arrhythmia presence (78). The LANDI-SEP trial confirmed effective heart rate reduction, achieving and maintaining target heart rate in 65.4% vs. 29.2% with placebo, without increased vasopressor requirements (79). In a landmark study, Morelli et al. showed that esmolol infusion in patients with septic shock and persistent tachycardia was associated with improved hemodynamic indices, although this trial focused on heart rate control rather than AF-specific outcomes (80).

In a meta-analysis, Sato et al. pooled all esmolol and landiolol RCTs in septic patients with persistent tachycardia (81). Overall 28-day mortality showed borderline significance (RR 0.83, 95% CI 0.68–1.00, *p* = 0.05), with single-center trials favoring beta-blockade but multicenter trials showing neutral or harmful effects. Trial sequential analysis confirmed insufficient evidence, highlighting the need for larger multicenter trials.

Alexandru et al. (2024) analyzed seven RCTs (*n* = 854) and found no mortality benefit from ultra-short acting beta-blockers (risk difference −0.10, 95% CI −0.22 to 0.02, *p* = 0.11; I^2^ = 73%) (82).

#### 5.5.4 Non-dihydropyridine calcium channel blockers

Non-dihydropyridine calcium channel blockers (diltiazem, verapamil) may be considered for rate control in patients with preserved left ventricular function (LVEF ≥ 35%) and significant atrial dilation (LAVi ≥ 40 mL/m^2^), where rhythm control is unlikely to succeed (Figure 1). The ESC 2024 guidelines recommend beta-blockers, diltiazem, verapamil, or digoxin as first-choice drugs for rate control in patients with AF and LVEF ≥ 40% (Class I, Level B) (19).

A comparative study of diltiazem vs. amiodarone for new-onset atrial arrhythmias in non-cardiac surgery ICU patients found similar rates of heart rate control, but diltiazem was associated with higher rates of treatment-limiting hypotension (83).

In clinical scenarios where beta-blockers are contraindicated or ineffective, cautious use of diltiazem may be considered in relatively hemodynamically stable patients with preserved left ventricular function, under close hemodynamic monitoring (3).

#### 5.5.5 Digoxin

Digoxin remains a useful but clearly second-line, predominantly adjunctive agent for rate control in septic patients with atrial fibrillation (AF), especially when heart failure with reduced ejection fraction (HFrEF) coexists (84, 85).

In critically ill patients, comparative data indicate that amiodarone achieves ventricular rate control more rapidly than digoxin, albeit at the expense of more bradycardia and greater vasopressor requirements (67). In this context, digoxin should be considered a slower-onset, hemodynamically favorable adjunctive rate-control agent. Contemporary observational evidence in AF, synthesized in an umbrella review of 12 meta-analyses, suggests that digoxin use is associated with a moderate increase in all-cause mortality and cardiovascular mortality (hazard ratio approximately 1.19 for each) (86). Conversely, high-quality randomized data in HFrEF (DIG and, more recently, DIGIT-HF) indicate that low-dose cardiac glycoside therapy does not increase mortality and reduces heart-failure hospitalizations when serum concentrations are kept in the lower therapeutic range (87, 88).

A recent large claims-based cohort of incident AF found no significant difference in the composite of in-hospital death or recurrent cardiovascular hospitalization between patients discharged on beta-blocker alone and those on digoxin alone or digoxin plus beta-blocker after robust propensity weighting (84). These findings, together with the RATE-AF study (89), suggest that when carefully dosed and monitored, digoxin can provide symptom and quality-of-life benefits without increased mortality risk.

In septic and other critically ill patients with NOAF, elevated sympathetic tone and frequent use of vasoactive drugs blunt the rate-control effect of digoxin and delay onset, making it unsuitable as a stand-alone first-line agent when rapid rate control is required. However, its favorable hemodynamic profile in ICU studies supports digoxin's use as an adjunctive rate-control agent in two specific clinical scenarios. First, in patients with heart failure with reduced ejection fraction (HFrEF), in whom further beta-blockade or non-dihydropyridine calcium channel blockade is precluded by hypotension or low cardiac output. Second, in patients with persistent tachycardia despite maximally tolerated doses of beta-blockers or amiodarone, adding digoxin may improve rate control without additional hemodynamic compromise (67, 85, 88–90).

#### 5.5.6 Vernakalant

Vernakalant is an atrial-selective antiarrhythmic agent that blocks multiple ion channels, with predominant effects on early-activating atrial potassium currents (Kv1.5/IKur) and frequency-dependent sodium channel blockade (91). In post-cardiac surgery and emergency department settings, conversion rates of 47%–76% within 90 min have been reported (91–93).

No randomized controlled trial, prospective study, or even case series has evaluated vernakalant specifically in patients with sepsis or septic shock. The feasibility of vernakalant use in the ICU was systematically assessed by Rudiger et al. (2014), who screened 191 post-cardiac surgery ICU patients with NOAF; only 32 (17%) met the strict eligibility criteria. The primary reason for exclusion was hemodynamic instability (94).

Given the complete absence of sepsis-specific evidence, the broad hemodynamic contraindications (SBP < 100 mmHg, vasopressor dependency, severe heart failure, recent IV antiarrhythmics), and the risk of transient but clinically significant hypotension even in relatively stable ICU patients, vernakalant cannot currently be recommended for AF management in sepsis or septic shock. A meta-analysis of nine RCTs (*n* = 1,421) confirmed that while vernakalant is effective for conversion of recent-onset AF (RR 6.61; 95% CI 2.78–15.71), it showed a non-significant trend toward increased hypotension (RR 1.51; 95% CI 0.62–3.68), and none of the included trials enrolled critically ill or septic patients (95).

### 5.6 Step 6: dynamic reassessment

Sepsis is characterized by rapidly evolving hemodynamics, and the dominant arrhythmia phenotype may shift over hours. A strategy appropriate at one time point may become harmful as the clinical picture changes. Continuous bedside reassessment - integrating heart rate and rhythm monitoring, hemodynamic parameters, and serial echocardiographic evaluation - is therefore essential. Treatment should be titrated, switched, or de-escalated based on the evolving clinical trajectory rather than applied as a fixed protocol. This aligns with the “E (Evaluation)” principle of the ESC AF-CARE framework, which emphasizes that dynamic reassessment should be individualized for every patient (19).

When cardioversion is performed, echocardiographic reassessment at 4 h provides critical prognostic information. Whilst pre-cardioversion parameters (LVEF, LAVi) guide the initial strategy, post-cardioversion functional indices (LAEF, AVTI) provide superior predictive ability for sinus rhythm maintenance and should inform the definitive decision between continued rhythm control and transition to rate control (55, 58). Left atrial emptying fraction (LAEF) greater than 38% and mitral A wave velocity-time integral (AVTI) greater than 7 cm at 4 h after cardioversion predict sustained sinus rhythm and support continuation of a rhythm-control strategy (Figure 1). Conversely, lower values, particularly LAEF below 20% or AVTI below 5 cm, indicate a high risk of recurrence and should prompt transition to a rate-control approach (58). Patients with sinus rhythm on ECG but without substantial mechanical atrial contraction (absent or minimal A wave on the mitral inflow pattern) are at particularly high risk of arrhythmia recurrence; in such patients, repeated attempts at cardioversion are unlikely to succeed and rate control should be preferred. Additionally, systolic pulmonary artery pressure (SPAP) ≥ 51 mmHg at 4 h post-cardioversion is associated with multiple AF recurrences and may necessitate a shift in management focus toward right ventricular unloading and optimization of pulmonary vascular resistance (55).

This approach emphasizes individualized, physiology-driven management rather than uniform protocols. By integrating hemodynamic phenotyping, systematic correction of reversible triggers, echocardiography-guided strategy, and continuous dynamic reassessment, the proposed algorithm provides a bedside-applicable framework for the management of supraventricular arrhythmias in sepsis and septic shock.

## 6 Anticoagulation considerations

Decisions about anticoagulation in critically ill patients with AF require balancing potential stroke-prevention benefits against a high baseline risk of hemorrhage in a population with multiple predisposing factors for bleeding. Conventional stroke-risk tools such as CHA_2_DS_2_-VASc were developed and validated in stable outpatient cohorts and may have limited accuracy for predicting thromboembolic risk in sepsis and ICU populations. Similarly, bleeding-risk scores like HAS-BLED have not been specifically validated in septic shock and may underestimate risk of bleeding in patients with sepsis-induced coagulopathy (96–98). Sepsis-induced coagulopathy (SIC) is characterized by concurrent procoagulant activation, consumptive thrombocytopenia, and antithrombin depletion. This further complicates anticoagulation decisions, as standard bleeding-risk assessments may substantially underestimate the true hemorrhagic risk in this population. Notably, SIC has itself been independently associated with the development of new AF episodes in ICU patients admitted in sinus rhythm (31% vs. 15.6% in SIC-negative patients, *P* < 0.001), creating a clinical paradox in which the coagulopathy increases AF risk (99).

Observational data confirm substantial practice variation: many clinicians defer systemic anticoagulation during the acute phase of critical illness, particularly in patients with septic shock, coagulopathy, or recent surgery given the absence of demonstrated in-hospital stroke reduction with parenteral anticoagulation during sepsis. In the largest available cohort (*n* = 38,582), Walkey et al. found that anticoagulation did not reduce ischemic stroke risk in either newly diagnosed or pre-existing AF. In the NOAF subgroup, anticoagulation was not associated with significantly increased bleeding (RR 0.97; 95% CI 0.83–1.14), whereas in patients with pre-existing AF, bleeding risk was significantly increased (RR 1.23; 95% CI 1.10–1.36; *P* for interaction=0.008) (96). The absence of both benefit (stroke reduction) and clear harm (bleeding) in the NOAF subgroup underscores the equipoise that persists around this question, rather than providing a definitive argument against anticoagulation (100).

Reflecting this uncertainty, the 2023 SSAI clinical practice guideline on NOAF in critically ill adults, based on very low-certainty evidence, issues a weak recommendation against routine use of therapeutic-dose systemic anticoagulation for NOAF and emphasizes individualized decision-making together with post-ICU cardiology follow-up (53). In clinical practice, a reasonable approach is to defer full-dose anticoagulation during the acute unstable phase of septic shock in the absence of clear thromboembolic indications, with reassessment once hemodynamic stability and bleeding risk improve. A complex echocardiography protocol may help to tailor anticoagulation strategy in critically ill patients (58).

Although sepsis-induced NOAF often appears transient during the acute illness, accumulating cohort data indicate high rates of AF recurrence (exceeding 50% over 5 years) and a persistent, approximately two-fold increase in long-term ischemic stroke risk compared with sepsis survivors without AF. Consequently, even when sinus rhythm is restored, decisions about long-term anticoagulation should be revisited after ICU discharge. Careful consideration of risk of AF recurrence and conventional stroke-risk factors should be assessed, rather than assuming that sepsis-related AF is benign and self-limited (97, 101).

Post-discharge cardiology follow-up with structured reassessment, including ambulatory rhythm monitoring and re-evaluation of CHA_2_DS_2_-VASc score once sepsis-related confounders have resolved, should be part of the standard care pathway for sepsis survivors with NOAF. No completed randomized trial has addressed anticoagulation specifically in sepsis-associated NOAF, and prospective data are urgently needed to define the optimal agent, timing, and duration in critically ill patients with sepsis.

## 7 Discussion

The key shift in the management of supraventricular arrhythmias in sepsis is from drug-centered care to phenotype- and echocardiography-guided therapy. This approach offers three contributions to the existing literature on supraventricular arrhythmias in sepsis. First, it introduces a phenotype-based classification (hemodynamically unstable, compensatory tachycardia, maladaptive adrenergic) that aligns arrhythmia management with the dominant hemodynamic mechanism rather than treating all supraventricular arrhythmias as a single entity. Second, it proposes a pragmatic, echocardiography-guided treatment algorithm (Figure 1) that translates the PRASE trial findings, particularly the LAVi-stratified treatment effect, into a structured bedside decision tool applicable across the spectrum of hemodynamic presentations. Third, it provides an updated synthesis of the beta-blocker, vernakalant, and anticoagulation evidence that highlights the fragility of current data and the need for phenotype-enriched trials.

The neutral results of the large study by Goulden et al., which evaluated magnesium supplementation around relatively low institutional thresholds (1.6–2.0 mg/dL; ≈0.66–0.82 mmol/L), should not be interpreted as evidence against correcting clear hypomagnesemia in septic patients (51). In PRASE, routine magnesium substitution led to a median level of 1.15 mmol/L of plasmatic total magnesium, without correction for ionized magnesium or verification of the very frequent intracellular deficit by a magnesium loading test. It is plausible that many patients considered “borderline low” in the JAMA cohort would still have been classified as overtly hypomagnesemic in other clinical settings (54). Current data suggest that routine supplementation may still provide some additional antiarrhythmic benefit, especially in cases of substantial intracellular magnesium deficits, which remain difficult to assess in clinical practice.

The PRASE trial remains the only double-blind randomized trial specifically comparing antiarrhythmic agents for supraventricular arrhythmias in septic shock, and its findings fundamentally reframe how rhythm-control decisions should be made in this population. Rather than supporting a single universal first-line agent, PRASE demonstrates that antiarrhythmic drug selection must be individualized based on left atrial size assessed by bedside echocardiography. Propafenone is the preferred agent in patients with non-dilated left atria, while amiodarone remains appropriate and may be safer when left atrial dilatation is present (54, 56). This echocardiography-guided, personalized approach represents a meaningful departure from the historical default of amiodarone for all critically ill patients with AF, and positions LAVi measurement as a routine pre-treatment step in ICU rhythm management. The biological plausibility of LAVi as a treatment-effect modifier is supported by independent evidence: a systematic review and meta-analysis by Raniga et al. demonstrated that LAVi < 40 mL/m^2^ is independently associated with significantly higher rates of sinus rhythm maintenance after direct current cardioversion, confirming that left atrial remodeling determines the substrate available for successful and durable rhythm control (102).

In contrast, the beta-blocker literature in sepsis is strikingly heterogeneous (77–80, 103). The single-center, open-label esmolol trial by Morelli et al. reported an impressive reduction in 28-day mortality in septic shock, which fueled enthusiasm for heart-rate control as a prognostic intervention. However, subsequent, more rigorously designed multicenter landiolol trials have not replicated this survival benefit. J-Land 3S showed that landiolol is effective for achieving target heart rate but was underpowered for mortality. The STRESS-L study, conducted in a more severely ill and vasopressor-dependent population, was stopped early for futility with respect to organ-failure scores and showed a concerning trend toward higher mortality in the landiolol arm. LANDI-SEP, enrolling a less severely ill cohort, confirmed that landiolol can safely achieve short-term heart-rate control but again without any impact on survival. The trial sequential analysis by Sato et al. confirms that the cumulative evidence for beta-blocker-associated mortality reduction in sepsis remains below the required information size, meaning that the current data are insufficient to confirm or refute a true survival benefit, and further adequately powered trials are needed (81). These findings collectively suggest that beta-blockers in sepsis should be reserved for carefully selected patients, primarily those with the maladaptive adrenergic phenotype (Section 3.3), and titrated cautiously under continuous hemodynamic monitoring.

The pragmatic treatment algorithm proposed in this review (Figure 1) is intended as a clinically applicable decision framework rather than a rigid protocol. Its structure - sequential assessment of hemodynamic stability, ventricular function, and left atrial size to guide both the rate-vs.-rhythm decision and the selection of specific pharmacological agents - reflects the convergence of the PRASE trial data, the echocardiographic framework developed from subsequent analyses (55, 58), and the phenotype-based approach increasingly advocated in sepsis resuscitation (20, 21).

The algorithm should be viewed as a testable hypothesis and echocardiographic thresholds require prospective validation in multicenter settings before they can be adopted as standard practice. Importantly, the iterative nature of the algorithm reflected in the continuous reassessment principle acknowledges that hemodynamic phenotypes in sepsis are dynamic and that the optimal strategy may change as the clinical picture evolves.

### 7.1 Limitations

This narrative review does not employ formal systematic methodology; the evidence synthesis is therefore qualitative and subject to the inherent selection and interpretation biases of this approach. No formal grading of evidence certainty (e.g., GRADE) was applied to individual recommendations, and the strength of the underlying data varies substantially across the topics addressed.

Many of the included studies were not conducted exclusively in septic populations, requiring extrapolation of findings to patients with sepsis and septic shock. The beta-blocker trials (J-Land 3S, STRESS-L, LANDI-SEP) were designed primarily for persistent sinus tachycardia rather than atrial fibrillation, and analyses of NOAF subgroups should be interpreted as hypothesis-generating rather than definitive. The PRASE trial, although the most relevant arrhythmia-specific RCT in septic shock, was a two-center study, and its findings particularly the LAVi-stratified subgroup analysis require external validation in multicenter settings.

The proposed treatment algorithm (Figure 1) is a pragmatic, expert-derived framework informed by the available evidence but has not been prospectively validated. Its implementation requires bedside echocardiographic expertise, including LAVi measurement and assessment of post-cardioversion atrial function (LAEF, AVTI), which may not be universally available in all ICU settings. The clinical phenotype classification (Section 3) is conceptual and has not been validated against objective hemodynamic or biomarker criteria.

Finally, regional differences in drug availability (e.g., landiolol is not licensed in North America; intravenous propafenone availability varies across Europe), and local echocardiographic expertise may limit the generalizability of the proposed algorithm. The anticoagulation recommendations are based entirely on observational data and expert opinion, with no completed RCT addressing this question in sepsis-associated NOAF.

## 8 Conclusion

Supraventricular arrhythmias, predominantly atrial fibrillation, are frequent and clinically relevant in sepsis, and recent trials support a shift toward echocardiography-guided, individualized management rather than uniform protocols.

The findings support two practical conclusions. First, for rhythm control in septic shock, antiarrhythmic drug selection should be guided by echocardiographic assessment of systolic ventricular function and left atrial size. Propafenone appears preferable to amiodarone in patients with LVEF ≥ 35% and non-dilated atria, whereas amiodarone is likely safer when severe systolic dysfunction or significant atrial remodeling are present. Second, ultra-short acting beta-blockers should be used as individualized tools for rhythm or heart-rate control in patients carefully selected with the aid of echocardiography and with adequate hemodynamic reserve, rather than as routine mortality-modifying therapy in sepsis. Robust data on mortality effects of beta-blockade and on anticoagulation in sepsis-related AF are still lacking, so future research must clarify which phenotypes benefit from rate or rhythm control strategies and anticoagulation, while current practice should remain guided by hemodynamic status and focused echocardiographic assessment.

## Author contributions

TT: Conceptualization, Investigation, Writing – original draft, Writing – review & editing. PW: Conceptualization, Formal analysis, Writing – original draft, Writing – review & editing. MB: Conceptualization, Supervision, Writing – original draft, Writing – review & editing.
